# Identification of Intact High Molecular Weight Glutenin Subunits from the Wheat Proteome Using Combined Liquid Chromatography-Electrospray Ionization Mass Spectrometry

**DOI:** 10.1371/journal.pone.0058682

**Published:** 2013-03-08

**Authors:** Bert Lagrain, Markus Brunnbauer, Ine Rombouts, Peter Koehler

**Affiliations:** 1 German Research Center for Food Chemistry, Freising, Germany; 2 Laboratory of Food Chemistry and Biochemistry and Leuven Food Science and Nutrition Research Centre (LFoRCe), Katholieke Universiteit Leuven, Leuven, Belgium; California Institute of Technology, United States of America

## Abstract

The present paper describes a method for the identification of intact high molecular weight glutenin subunits (HMW-GS), the quality determining proteins from the wheat storage proteome. The method includes isolation of HMW-GS from wheat flour, further separation of HMW-GS by reversed-phase high-performance liquid chromatography (RP-HPLC), and their subsequent molecular identification with electrospray ionization mass spectrometry using a quadrupole-time-of-flight mass analyzer. For HMW-GS isolation, wheat proteins were reduced and extracted from flour with 50% 1-propanol containing 1% dithiothreitol. HMW-GS were then selectively precipitated from the protein mixture by adjusting the 1-propanol concentration to 60%. The composition of the precipitated proteins was first evaluated by sodium dodecyl sulfate-polyacrylamide gel electrophoresis with Coomassie staining and RP-HPLC with ultraviolet detection. Besides HMW-GS (≥65%), the isolated proteins mainly contained ω5-gliadins. Secondly, the isolated protein fraction was analyzed by liquid chromatography-mass spectrometry. Optimal chromatographic separation of HMW-GS from the other proteins in the isolated fraction was obtained when the mobile phase contained 0.1% trifluoroacetic acid as ion-pairing agent. Individual HMW-GS were then identified by determining their molecular masses from the high-resolution mass spectra and comparing these with theoretical masses calculated from amino acid sequences. Using formic acid instead of trifluoroacetic acid in the mobile phase increased protein peak intensities in the base peak mass chromatogram. This allowed the detection of even traces of other wheat proteins than HMW-GS in the isolated fraction, but the chromatographic separation was inferior with a major overlap between the elution ranges of HMW-GS and ω-gliadins. Overall, the described method allows a rapid assessment of wheat quality through the direct determination of the HMW-GS composition and offers a basis for further top-down proteomics of individual HMW-GS and the entire wheat glutenin fraction.

## Introduction

Gluten proteins or prolamins are the storage proteins of wheat (*Triticum aestivum* L.) and represent an important fraction of the daily human protein intake. They are insoluble in water, but can be divided into alcohol soluble gliadins and alcohol insoluble glutenins. Wheat gluten proteins are characterized by their ability to form a cohesive viscoelastic mass when mixed with water. Because of their unique properties, they play a key role in the appearance and quality of different wheat-based foods, such as bread, pasta and pastry [1].

Although end-use quality of common wheat is influenced by growing conditions and genotype, the composition of glutenin is responsible for the major part of the variability in wheat quality. Glutenin is a polymeric protein consisting of disulfide linked glutenin subunits (GS) [1]. Structurally GS can be grouped into low-molecular-weight (LMW)- and high-molecular-weight (HMW)-GS. As such, the composition of the HMW-GS alone may account for up to 60% variation in the quality of bread flour [2]. This underlines the importance of a correct and reliable detection of the HMW-GS composition of a given wheat cultivar.

Investigation of prolamins by current methodologies is often challenging due to the limited genome information of cereals [3] and their poor solubility. The allelic composition of HMW-GS (mostly three to five protein components per cultivar) is typically distinguished by sodium dodecyl sulfate-polyacrylamide gel electrophoresis (SDS-PAGE), which separates proteins based on their apparent molecular mass [4]. SDS-PAGE also led to the current HMW-GS nomenclature, in which individual subunits are numbered in order of increasing mobility on the gel [4]. However, on SDS-PAGE the HMW-GS show anomalously high relative molecular masses (M_r_s) ranging from 80,000 to 120,000, while, according to known amino acid sequences, they have M_r_s ranging from 65,000 to 90,000. In addition to the overestimated M_r_ of all HMW-GS, the relative mobilities of individual subunits in SDS-PAGE are not always directly related to differences in their M_r_ [5]. Therefore, other techniques, such as high-performance liquid chromatography (HPLC) and lab-on-a-chip capillary electrophoresis, but also mass spectrometry (MS), have been developed to identify and characterize HMW-GS [6], [7]. Especially, matrix-assisted laser desorption/ionization time-of-flight (MALDI-TOF) MS has proven to be a sensitive technique to determine the molecular weights of intact HMW-GS [7]. At first it was difficult to distinguish mixtures of HMW-GS in a single MALDI-TOF analysis due to suppression phenomena resulting in poor resolution [8], but finally the molecular weights of different HMW-GS were determined with reasonable accuracy [7], [9], [10].

While MALDI-TOF MS is often used for direct identification of (simple) protein mixtures, which is the case for isolated HMW-GS, or in combination with two-dimensional gel electrophoresis, electrospray ionization (ESI)-MS is a liquid-based method and is compatible with typical chromatographic separations of biosamples. ESI produces a range of charged species for each biomolecule, which increases the precision of mass assignments [11], [12]. For gluten and other proteins, most applications for ESI-MS involve protein identification by proteolytic digestion followed by liquid chromatography and tandem MS of individual ions from the resulting peptide mixture. This ‘bottom-up’ or ‘shotgun’ approach has been used to verify HMW-GS sequences as derived from their genes [13], [14], wheat gluten composition [15], but also gluten structures [16]. The bottom-up approach becomes challenging with increasing complexity of the protein mixture. As a result, the identified peptides sometimes only represent a part of the full protein sequence, low MW proteins are not always detected, and there is a potential loss of data regarding isoforms and post-translational modifications [11], [17], [18]. In addition, wheat prolamins, and especially HMW-GS, are large proteins and have long repetitive sequences with few tryptic cleavage sites, leading to a peptide pool with unfavorable MS/MS characteristics [3]. Hence, a ‘top-down’ approach, in which intact proteins are measured and (partially) sequenced, can be advantageous for primary structure determination and the detection of specific protein modifications [11]. For top-down proteomics ESI is preferred over MALDI as ion source, because mono-charged protein ions, such as those generated by a MALDI source, cannot be detected with high resolution [18]. Furthermore, in order to achieve the full potential of top-down approaches, the chromatographic separation of intact proteins should be brought to the level as now achieved in shotgun proteomics for routine tandem MS analysis [19]. Reversed-phase (RP-) HPLC has been widely applied to cereal proteins and has proven to be a highly efficient tool for the qualitative and quantitative investigation and isolation of intact gliadins and GS [20], [21], [22]. RP-HPLC has been used in combination with ESI-MS to identify gliadins and LMW-GS [23], [24]. Nevertheless, the detection of intact HMW-GS with ESI-MS has not yet been reported, despite their key role in glutenin structure, wheat differentiation and quality. Proteomics of wheat, including a reliable detection of HMW-GS, combined with a full transcriptome analysis would offer an effective approach for controlling the genetic improvement of wheat [25]. A top-down approach using LC-ESI-MS can reveal the transcriptome protein structure including the positioning of post-translational modifications [26]. Given their unique and high masses, a direct, accurate and sensitive detection of HMW-GS can also be used to monitor wheat contamination in foods, which is necessary for people on a gluten-free diet.

Earlier studies using LC-ESI-MS to identify intact gluten proteins, focused on isolated LMW-GS only [24] or entire gliadin and reduced glutenin fractions [23]. In the latter study, different gliadins and LMW-GS could be distinguished, but at the elution times of the HMW-GS no distinct molecular mass could be obtained [23]. Furthermore, it was reported that, unlike gliadin, the components of glutenin show a greater mass variability, which impeded comparison of proteins from different wheat cultivars [23]. Therefore, the aim of this study was first to isolate commonly occurring HMW-GS from flour to reduce the high complexity of a reduced glutenin mixture in LC-ESI-MS [23]. Then specific conditions for HMW-GS separation and identification using RP-HPLC-ESI-MS were determined.

## Materials and Methods

### Wheat Flour

Three common wheat cultivars (cvs.) with known HMW-GS composition were selected: Akteur (subunits Ax1, Bx7, By9, Dx5, Dy10), Contra (Bx6, By8, Dx2, Dy12) and Apache (Ax2*, Bx7, By9, Dx3, Dy12). Wheat kernels of the cv. Akteur (harvest 2009) and Apache (harvest 2011) were milled into white flour with a Bühler Mill (Bühler, Uzwil, Switzerland). Wheat kernels of the cv. ‘Contra’ (harvest 2009) were milled into white flour with a Quadrumat Junior Mill (Brabender, Duisburg, Germany). All flour samples were sifted through a 0.2 mm screen.

### Chemicals and Reagents

The quality of all chemicals was of analytical grade or stated otherwise. Acetonitrile (ACN, LiChrosolv), Coomassie Brillant Blue R-250, formic acid (98–100%), glacial acetic acid, hydrochloric acid (32%, w/w), methanol (LiChrosolv), 1-propanol (LiChrosolv), sodium azide, SDS, trichloroacetic acid, and tris(hydroxymethyl)aminomethane (Tris) were from Merck (Darmstadt, Germany). Calcium chloride hexahydrate, sodium hydroxide (≥98%), and trifluoroacetic acid (TFA, ≥98%) were from Sigma-Aldrich (Steinheim, Germany). 3-(N-morpholino)propanesulfonic acid (MOPS) was from AppliChem (Darmstadt, Germany). Dithiothreitol (DTT), ethylenediaminetetraacetic acid (EDTA), phenol red, and serva blue G250 were from Serva (Heidelberg, Germany). Water was deionized by a Millipore-O Milli-Q purification system (Merck Millipore, Billerica, MA, USA).

### Determination of Flour Protein Content

Flour protein contents were determined in triplicate, using an adaptation of the AOAC Official Method 990.03 [27] to an automated Dumas protein analysis system (EAS variomax N/CN, Elt, Gouda, The Netherlands). A conversion factor of 5.7 was used to calculate protein from nitrogen content.

### Determination of Flour Protein Composition

Flour protein compositions, and levels of ω5-gliadin and HMW-GS in particular, were determined by an adapted Osborne fractionation according to Wieser et al. [22]. Hereto, flour samples (100.0 mg) were extracted twice with 1.0 ml of a salt solution (0.4 mol/L NaCl, 0.067 mol/L HKNaPO_4_ pH 7.6) (yielding the albumin/globulin extract), three times with 0.5 ml 60% (v/v) ethanol (yielding the gliadin extract) and twice with 1.0 ml 0.05 mol/L Tris/HCl buffer (pH 7.5) containing 50% 1-propanol, 2.0 mol/L urea and 1.0% (w/v) DTT and kept under nitrogen (yielding the reduced glutenin extract). Protein extracts were then subjected to RP-HPLC with a Jasco X-LC system (Jasco, Easton, MD, USA). After filtration over polyethersulfone (0.45 µm), protein extracts were loaded (10 µL) on an Acclaim 300 C_18_ column (Dionex, Düren, Germany). The elution system consisted of deionized water (A) and ACN (B), both with 0.1% TFA (v/v). Between analyses, the column was equilibrated at 24% B. For each run the following gradient was used based on Wieser et al. [22], [28]: (i) isocratic at 0% B for 0.4 min; (ii) linear from 0 to 24% B over 0.1 min; (iii) linear from 24 to 56% B over 19.5 min; (iv) isocratic at 90% B for 4 min; (iv) linear from 90 to 0% B in 0.1 min; (v) isocratic at 0% B for 5.8 min. Proteins were eluted (60°C, flow rate: 0.2 mL/min) and detected at 210 nm.

α-Gliadin, γ-gliadin, ω5- and ω1,2-gliadins, LMW-GS and HMW-GS were distinguished based on their elution order [22]. To determine the mass fraction of the different protein types, PWG-gliadin (Prolamin Working Group, Freising, Germany) was used as calibration reference [29].

### Isolation of HMW-GS Fractions from Flour

HMW-GS were extracted from flour according to Marchylo et al. [20]. Flour (33 g) was stirred for 30 min at 60°C in 200 mL 50% (v/v) 1-propanol containing 1.0% DTT (w/v). After centrifugation (20 min at 4600 *g*), the supernatant was collected and the residue was again extracted with 100 mL of the same solvent by stirring it for 30 min at 60°C. After centrifugation (20 min at 4600 *g*), the combined supernatants (300 mL) were filtered over paper. HMW-GS were then selectively precipitated at ambient conditions by gradually adding 75 mL 100% 1-propanol to bring the 1-propanol concentration of the supernatant to 60.0% (v/v). The suspension was allowed to stand at ambient conditions for 30 min. The precipitate was then collected after centrifugation (20 min at 4600 *g*) and removal of the supernatant. For further analysis by SDS-PAGE and RP-HPLC the precipitated proteins were freeze-dried. For LC-MS analysis, the collected precipitate was dried under vacuum with a rotational vacuum concentrator (Christ RVC 2–25, Martin Christ, Osterode am Harz, Germany) immediately after centrifugation.

### SDS-PAGE of Isolated HMW-GS Fractions

SDS-PAGE was carried out according to Lagrain et al. [5] based on the method of Kasarda et al. [30] with a homogeneous NuPAGE 10% polyacrylamide - Bis-Tris [Bis(2-hydroxyethyl)-amino-tris(hydroxymethyl)-methane-HCl] gel at pH 6.4, 1.0 mm×10 well (Invitrogen, Carlsbad, CA, USA). The running buffer was MOPS-Tris (50 mmol/L MOPS, 50 mmol/L Tris, 3.5 mmol/L SDS, 1 mmol/L EDTA, pH 7.7) containing DTT (5 mmol/L) as reducing agent added to the inside chamber. Freeze-dried HMW-GS (1.0 mg) were mixed with 1 mL of extraction buffer (293.3 mmol/L sucrose, 246.4 mmol/L Tris, 69.4 mmol/L SDS, 0.51 mmol/L EDTA, 0.22 mmol/L serva blue G250, 0.177 mmol/L phenol red, 0.105 mmol/L HCl, pH 8.5) for 24 h under reducing conditions (DTT, 50 mmol/L). The protein suspension was then shaken for 10 min at 60°C and centrifuged at 5000 *g* for 5 min at 20°C. Five proteins with different M_r_ (myosin, 200 k; β-galactosidase, 116 k; bovine serum albumin, 66 k; ovalbumin, 43 k; carbonic anhydrase, 29 k) were used as M_r_ markers. The sample volumes on the gel were between 5 to 10 µL per slot. Running time was 40 min at 200 V and 115 mA. After the run, proteins were fixed for 30 min in 12% (w/w) trichloroacetic acid and stained for 30 min with Coomassie Brilliant Blue R-250. Gels were first destained twice with methanol/water/acetic acid (50/40/10, v/v/v) for 15 min and then overnight with water/methanol/acetic acid (80/10/10, v/v/v).

### RP-HPLC of Isolated HMW-GS Fractions

Freeze-dried HMW-GS isolates were dissolved (20 min, 60°C) in 50% (v/v) 1-propanol containing 1.0% DTT (w/v) to obtain a concentration of 1.0 mg protein/mL. Protein solutions (10 µl) were then subjected to RP-HPLC (see above). Protein composition of the HMW-GS isolates was also determined according to Wieser et al. [22] and mass fractions of the different protein types in the HMW-GS isolates were determined with PWG-gliadin as calibration reference. Yields of protein types, in particular HMW-GS and ω5-type gliadins, were calculated by comparing their levels in flour to their levels in the HMW-GS isolate.

### LC-MS of Isolated HMW-GS Fractions

Vacuum-dried HMW-GS isolates were dissolved in 30% ACN containing 0.4% (v/v) TFA to a concentration of 1.0 mg protein/mL. LC-MS experiments were performed on an ESI-QTOF mass spectrometer (microTOF-Q, Bruker Daltonics, Bremen, Germany) coupled with an UltiMate 3000 HPLC (Dionex, Idstein, Germany) system equipped with an Xbridge BEH300 C_4_ 3.5 µm column (2.1×150 mm; Waters, Milford, MS, USA). The mobile phase for LC separation was (A) 0.1% (v/v) formic acid or TFA in water and (B) 0.1% (v/v) formic acid or TFA in acetonitrile. The same gradient was used as for regular RP-HPLC (see above). The flow rate was 0.2 mL/min, injection volume was 20 µL, and column temperature was 60°C. The mass spectrometer was operated in the positive mode (capillary voltage: −4000 V; end plate offset: −500 V). Nitrogen was used as drying (8.0 L/min, 180°C) and nebulizing gas (0.13 MPa). The scan range was *m/z* 750 to 2,500 (quadrupole ion energy: 5.0 eV). Analysis of the LC-MS data files was performed using Bruker Daltonics DataAnalysis software. M_r_ was calculated with related-ion deconvolution (mass range: 5,000–100,000, maximum charge: 100, minimum peaks in compound: 3, maximum number of compounds: 10, envelope cut-off: 75%, M_r_ agreement: 0.05%) and maximum entropy deconvolution (mass range: 5,000–100,000, instrument resolution power: 10,000).

## Results and Discussion

### Isolation and Composition of the HMW-GS Enriched Fraction

**Figure 1:**
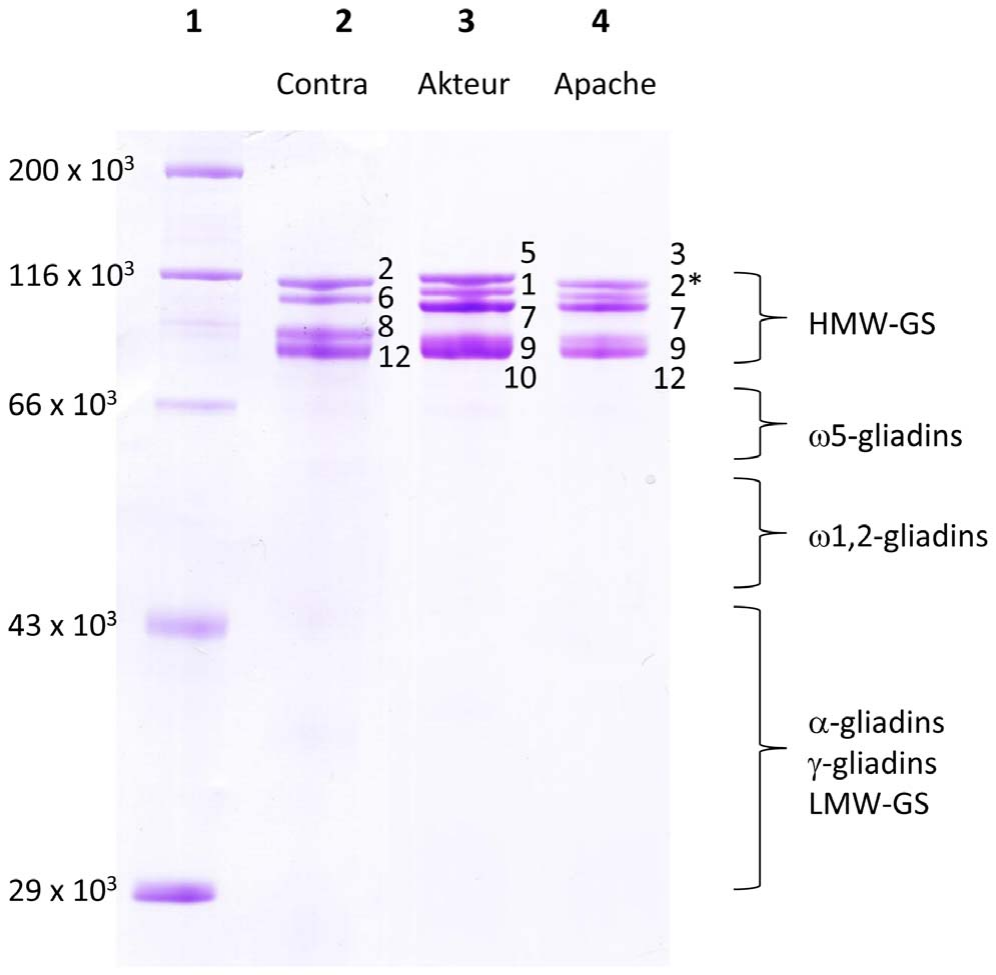
SDS-PAGE of isolated HMW-GS fractions from three wheat cultivars (cvs.). The subunit composition of wheat cvs. Contra (lane 2), Akteur (lane 3) and Apache (lane 4) is indicated with numbers according to the current nomenclature system [4]. Protein M_r_ markers are given in lane 1 (from top: myosin, β-galactosidase, bovine serum albumin, ovalbumin, carbonic anhydrase).

**Figure 2:**
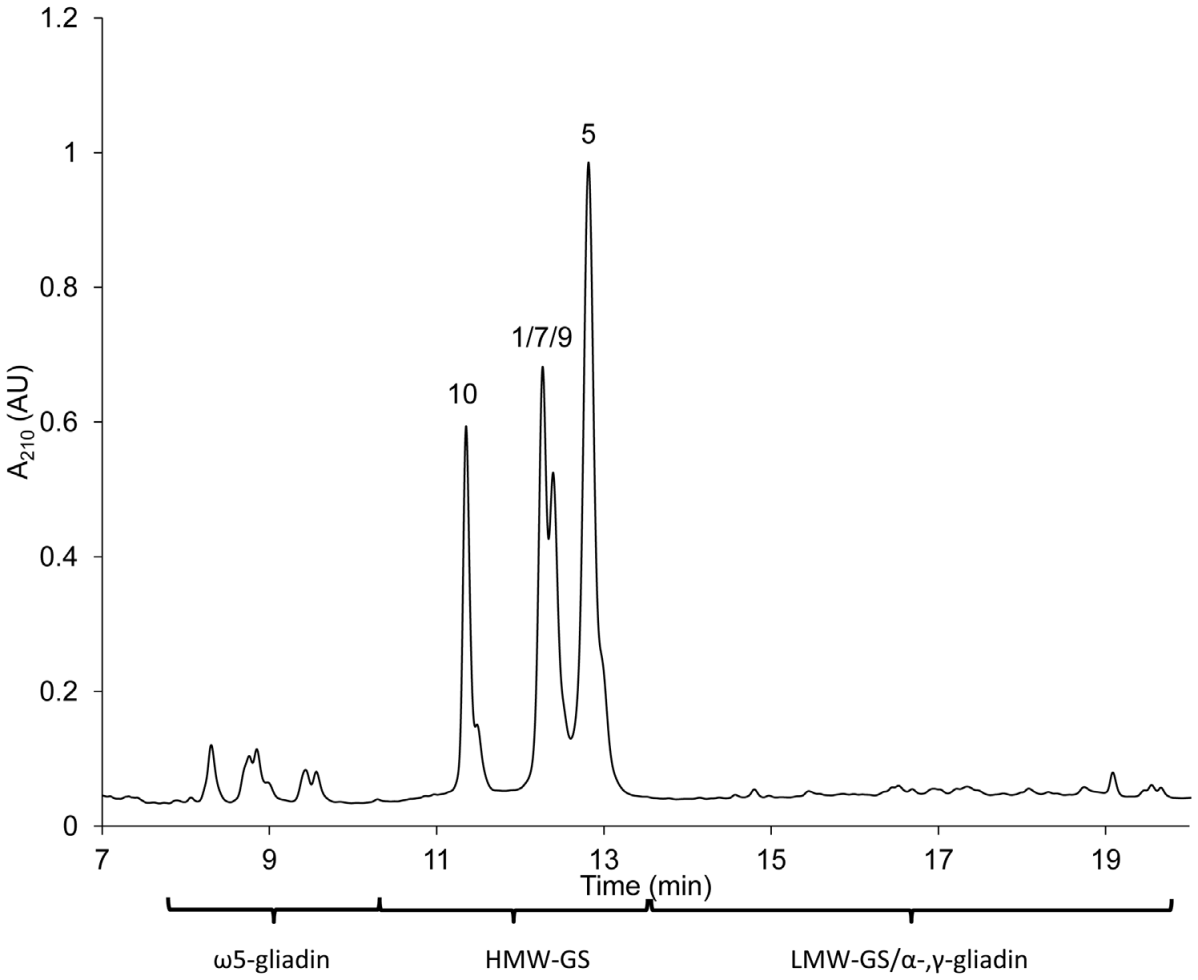
RP-HPLC chromatogram (210 nm) of the isolated HMW-GS fraction of wheat cv. Akteur. HMW-GS are labeled with numbers according to their current nomenclature system [4].

To reduce the complexity of a mixture of gluten proteins, HMW-GS were selectively isolated from wheat flour by their precipitation [20]. Figure 1 shows the Bis-Tris SDS-PAGE of the isolated proteins of cvs. Akteur, Contra and Apache that precipitated from the dissolved and reduced wheat proteins in 50% (v/v) 1-propanol containing 1% (w/v) DTT by adjusting the propanol concentration to 60% (v/v) at ambient conditions [20]. While classical Tris-glycine SDS-PAGE leads to anomalous migration orders of HMW-GS, the Bis-Tris SDS-PAGE system separates all HMW-GS according to their amino acid chain length [5]. In order to visualize the potential presence of proteins other than HMW-GS in the precipitate, a tenfold higher protein concentration compared to the one described in Lagrain et al. [5] was applied. Despite the high protein concentration on the gel, all subunits were well separated, except for subunits 9 and 10 in cv. Akteur. Both order and composition of HMW-GS from wheat cvs. Akteur, Contra and Apache were confirmed with the Bis-Tris gel shown in Figure 1. Furthermore, the protein pattern of the gel clearly indicated HMW-GS as the major protein fraction (Figure 1). To determine the quantity and yield of the proteins in the precipitated HMW-GS fraction, RP-HPLC was used according to Wieser et al. [22]. In this method proteins elute according to different surface hydrophobicity. This implies that, for gluten proteins, each protein type is separated as a unique subgroup and can be quantified without major overlap [22]. RP-HPLC analysis of the Osborne protein fractions of flour revealed that about 7% of all proteins in flour were HMW-GS (Table 1). The protein elution pattern from RP-HPLC of an isolated HMW-GS fraction (cv. Akteur) is given in Figure 2 and confirms HMW-GS as the most important fraction in the precipitated protein fraction. Calculation of protein levels in the HMW-GS fractions from their RP-HPLC chromatograms further illustrates the successful isolation of HMW-GS. At least 65% of the total mass of precipitated proteins consisted of HMW-GS. More than 90% of HMW-GS present in flour were recovered in the precipitate (Table 1). An important part of other gluten proteins that co-precipitated with the HMW-GS (about 15 to 25% of the total mass of precipitated proteins) consisted of ω5-gliadins, including the glutenin-bound (ωb-gliadins or D-LMW-GS) ω5-gliadins, while only trace amounts (<4%) of other (gluten) proteins precipitated. HMW-GS yields are consistent for the three wheat cultivars, but more proteins co-precipitated with the HMW-GS in the protein extract from cv. Contra than in the extracts from cvs. Akteur and Apache (Table 1). Overall, the above described method provided a fast, simple and efficient way to obtain relatively pure wheat HMW-GS fractions with high yields and suitable for further MS analysis.

**Table 1:** Levels of ω5-gliadins and HMW-GS in flour and isolated HMW-GS fractions of the wheat cvs. Akteur, Contra and Apache and the respective protein yields in the HMW-GS fractions.

	ω5-gliadin^1^	HMW-GS	Other wheat proteins
Wheat flour (% of total flour protein)			
Akteur	6.3	7.0	86.7
Contra	4.0	7.5	88.5
Apache	5.0	6.2	88.8
Isolated HMW-GS (%)			
Akteur	10.4	74.2	15.4
Contra	9.7	64.6	25.7
Apache	6.3	86.5	7.2
Protein yield in HMW-GS fraction (% of protein type in flour)			
Akteur	14.2	91.9	1.6
Contra	25.6	91.7	3.2
Apache	8.5	93.2	0.6

1 Including the glutenin-bound (ωb-) gliadins.

### LC-MS of the HMW-GS Enriched Fraction

**Figure 3:**
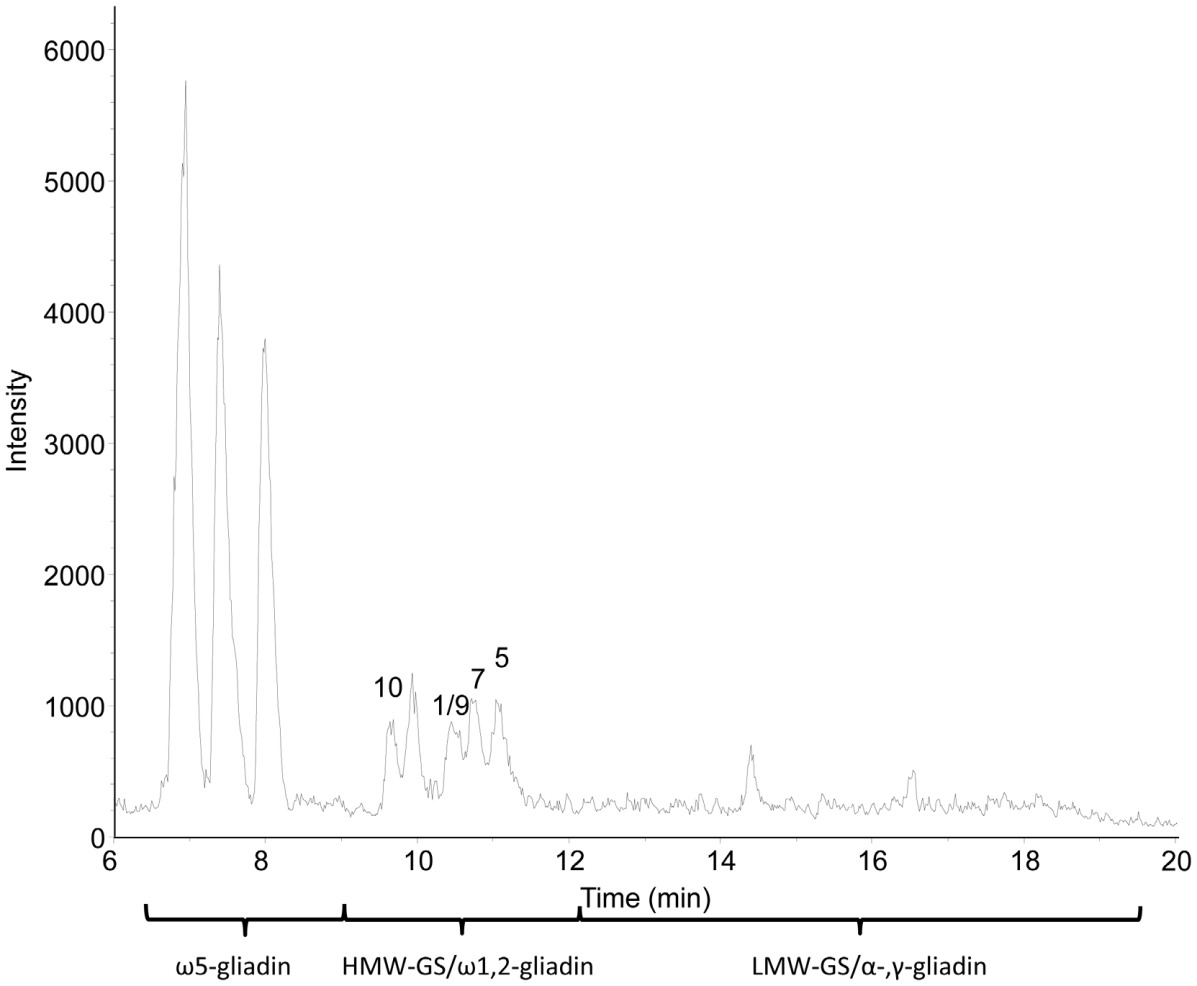
Base peak mass chromatogram from RP-HPLC-ESI-MS of the isolated HMW-GS fraction of wheat cv. Akteur. The mobile phase contains 0.1% (v/v) TFA. HMW-GS are labeled with numbers according to the current nomenclature system [4].

**Figure 4:**
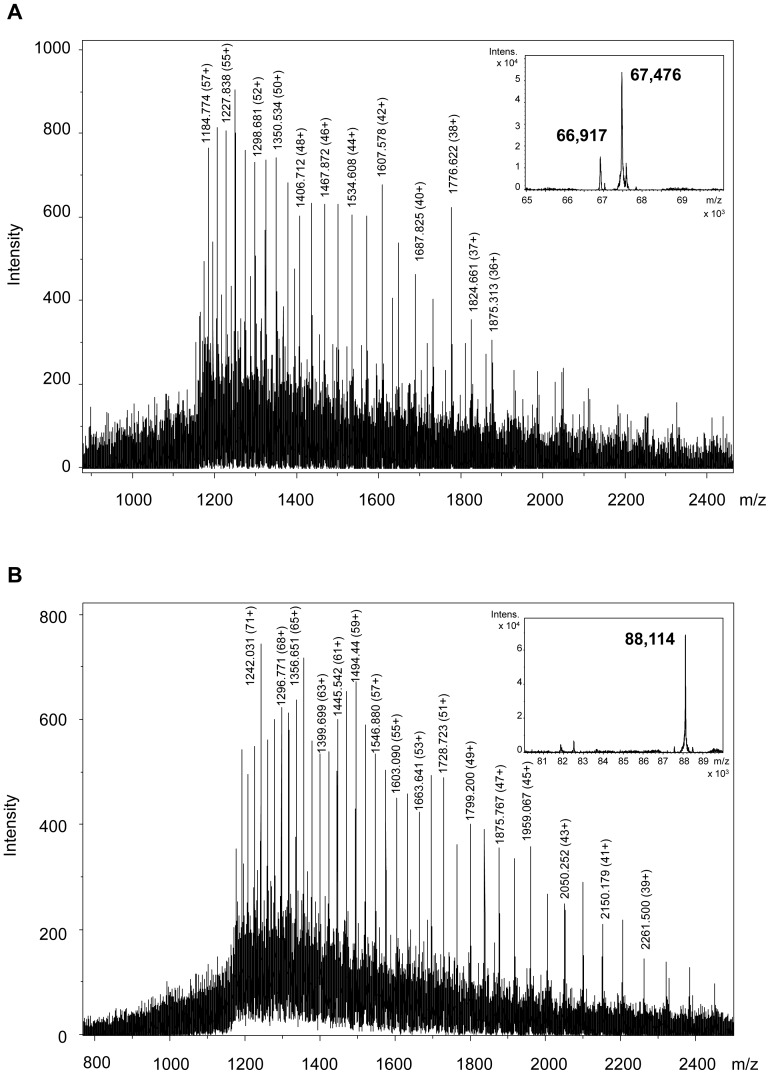
Mass spectra of wheat HMW-GS Dy10 (A) and Dx5 (B). The spectra are taken from the average of scans under the peaks with retention times 9.7 min (A) and 11 min (B) from the base peak mass chromatogram after RP-HPLC-ESI-MS of the isolated HMW-GS fraction from wheat cv. Akteur. The simulated mass spectra obtained by maximum entropy deconvolution are shown as insets.

A high-resolution ESI-QTOF instrument was used for LC-MS. No satisfactory LC-MS results were obtained when redissolved freeze-dried HMW-GS were used. Without reducing agent, the freeze-dried HMW-GS fraction did not dissolve well in organic solutions [50% (v/v) 1-propanol or 30% (v/v) ACN]. With reducing agent, the proteins did not result in identifiable HMW-GS peaks in the MS base peak chromatogram (BPC) in either of the organic solvents (results not shown). The latter was also reported by Mamone et al. when they analyzed redissolved freeze-dried glutenin reduced with DTT by LS-ESI-MS [23]. To avoid the risk of sample reoxidation and the subsequent need of a reducing agent for redissolving a freeze-dried sample, samples were vacuum-dried immediately after precipitation and supernatant removal. For ESI-MS analysis of proteins it is advantageous to dissolve them in an acidified mixture of water and an organic solvent [12]. Here, the protein fraction was dissolved in 30% (v/v) ACN containing 0.4% (v/v) TFA (1 mg/mL), because this particular sample preparation generated stable mass spectra in MALDI-TOF-MS [7]. The dissolved HMW-GS fraction was applied to LC-MS using a C_4_ column at 60°C and the same mobile phases, flow rate and gradient conditions as in regular RP-HPLC. A C_4_ column was chosen to avoid the risk of sample loss, and concomitant intensity loss in MS, as reported for C_18_ columns used for regular RP-HPLC [19]. The use of a C_4_ column indeed led to higher peak intensities compared to the standard C_18_ column (results not shown). The C_4_ column had a slightly negative effect on peak resolution compared to the standard column, but it provided a comparable protein pattern (Figures 2 and 3). Very low intensities were noted for the gliadin/LMW-GS fraction. Remarkably, high peak intensities were present for the ω5-gliadins, despite their low abundance. The opposite was observed for HMW-GS, which had rather low peak intensities in the BPC (Figure 3). This illustrates the complex relationship between the amount of protein present and its measured signal intensity, which is still poorly understood [31]. In these experimental conditions, the concentration of HMW-GS in the sample had to be at least 0.6 mg/mL (with an injection level of 20 µL) to obtain highly resolved mass spectra with a sufficient intensity for deconvolution. A protein appeared as a cluster of multiply charged ions (Figure 4), from which the M_r_ was calculated by determining the charge state of each signal (related-ion deconvolution, Table 2). Additionally, protein M_r_ was also determined with maximum entropy deconvolution, which is based on a mathematical algorithm for subtracting electronic noise and calculating the most probable molecular weight [32]. The result is then presented as a simulated mass spectrum (insets in Figure 4). Both deconvolution techniques resulted in the same M_r_ for each protein. For cvs. Akteur and Apache, eight peaks could be distinguished in the BPC which represented nine major proteins in each cultivar (Figure 3, Table 2). For cv. Contra seven peaks represented eight proteins (Table 2). All HMW-GS of cvs. Akteur, Contra and Apache were identified with very high accuracy, by comparing the M_r_ of the proteins in the BPC with the M_r_ of the HMW-GS as calculated from their amino acid sequences (Table 3). In general, the average M_r_s obtained in this study by LC-ESI-MS agreed better with the molecular masses calculated from the known amino acid sequences in comparison to those obtained with MALDI-TOF MS by Gao et al. [9]. The strong agreement between measured and calculated M_r_ further implies that HMW-GS underwent little if any post-translational modifications, which confirms previous research [33], [34]. For subunit Dx3 from common wheat no amino acid sequence has been reported in literature. It was shown earlier that Dx3 shows sequence homologies with Dx subunits from *Aegilops tauschii* (Tausch's goatgrass), which has contributed the D genome in wheat [5], [9]. However, the M_r_ of Dx3 found in this study (87,207) clearly differs from the protein M_r_s calculated from the *Dx3^t^* (87,655) and *Dx4^t^* (86,666) genes of *Aegilops tauschii* [35]. Thus, the unique primary structure of wheat Dx3 remains to be elucidated. Most HMW-GS eluted as single peaks in the BPC, except for subunits 1 or 2* and 9 on the one hand; and 6 and 12 on the other hand. Besides the M_r_s reported in Table 3 for cv. Akteur, also proteins with M_r_ 66,917 (Figure 4A) and 81,871 were detected with lower intensities at retention times (RTs) 9.7 and 10.7 min, respectively. Most likely, these proteins had the same sequences as subunits 10 and 7 except from a deletion of about 6 amino acids in the C-terminal part, as reported previously [13], [14].

**Table 2:** Relative average molecular masses (M_r_) and corresponding proteins detected in the isolated HMW-GS fractions of the cvs. Akteur, Contra and Apache as determined by RP-HPLC-ESI-MS with the mobile phase containing 0.1% (v/v) TFA.

Wheat cultivar	RT (min)	M_r_ 1	Identification^2^
Akteur			
	6.9	54,838 (0.7)	ω5-gliadin
	7.4	54,951 (0.5)	ω5-gliadin
	8.0	51,527 (0.4)	ω5-gliadin
	9.7	67,476 (0.6)	HMW-GS Dy10
	9.9	41,863 (0.6)	ω1,2-gliadin
	10.4	87,697 (0.7)	HMW-GS Ax1
		73,519 (0.5)	HMW-GS By9
	10.7	82,528 (0.7)	HMW-GS Bx7
	11.0	88,114 (0.7)	HMW-GS Dx5
Contra			
	7.0	54,836 (0.6)	ω5-gliadin
	7.5	51,413 (0.5)	ω5-gliadin
	8.1	51,526 (0.7)	ω5-gliadin
	9.7	68,512 (0.8)	HMW-GS Dy12
		86,477 (0.6)	HMW-GS Bx6
	10.1	41,862 (0.7)	ω1,2-gliadin
	10.5	75,239 (0.8)	HMW-GS By8
	11.2	87,105 (0.7)	HMW-GS Dx2
Apache			
	6.9	54,837 (0.5)	ω5-gliadin
	7.4	54,952 (0.6)	ω5-gliadin
	8.0	51,528 (0.6)	ω5-gliadin
	9.6	68,532 (0.7)	HMW-GS Dy12
	10.0	41,863 (0.6)	ω1,2-gliadin
	10.4	86,338 (0.6)	HMW-GS Ax2*
		73,521 (0.7)	HMW-GS By9
	10.7	82,529 (0.7)	HMW-GS Bx7
	11.0	87,207 (0.6)	HMW-GS Dx3

1 Standard deviations are given between brackets.

2 Based on M_r_ agreement with proteins from the wheat storage proteome [5], [36].

**Table 3:** Comparison of the theoretical relative molecular mass (M_r_) of HMW-GS calculated from their known amino acid sequences with their corresponding M_r_ determined by RP-HPLC-ESI-MS.

HMW-GS	Calculated M_r_ 1	M_r_ by ESI-MS	Difference	
Ax1	87,678	87,697		−19
Ax2*	86,335	86,338		−3
Bx6	86,393	86,477		−84
Bx7	82,526	82,528		−2
By8	75,131	75,239		−108
By9	73,517	73,519		−2
Dx5	88,126	88,114		12
Dx3	−2	87,207		−
Dx2	87,007	87,105		−98
Dy12	68,713	68,512		201
Dy10	67,474	67,476		−2

1 Based on results by Lagrain et al. [5].

2 No gene or protein sequence has been reported for Dx3.

The MS method appeared to be particularly sensitive for ω-gliadins. Besides the high intensities of the ω5-gliadins (Figure 3), also a protein with M_r_ 41,863 was observed co-eluting with the HMW-GS, although visual inspection of the SDS-PAGE gel (Figure 1) or the RP-HPLC chromatogram measured at 210 nm (Figure 2) showed no such proteins. Based on its M_r_ and RT, the protein was identified as ω1,2-gliadin, probably glutenin-bound [22], [36]. Again, very small amounts of protein elicited higher intensities than the far more abundant HMW-GS.

The rather low signal intensities of HMW-GS in the BPC compared to other gluten proteins, such as ω-gliadins, despite their abundance in the sample, might explain the previous difficulties in their detection with ESI-MS from a reduced glutenin sample [23]. Moreover, the used mobile phases, which resulted in an optimal separation in RP-HPLC, contained the strong acid TFA. The latter is known as a strong ion-pairing agent that decreases the ion yield and suppresses the MS signal, which leads to lower responses for analytes compared to formic acid in the mobile phase [37], [38]. The next section addresses the effect of formic acid in the mobile phase on the chromatographic separation and MS intensities of the proteins in an isolated HMW-GS fraction.

### Effect of TFA Versus Formic Acid on the Chromatographic Separation and Mass Intensities

**Figure 5:**
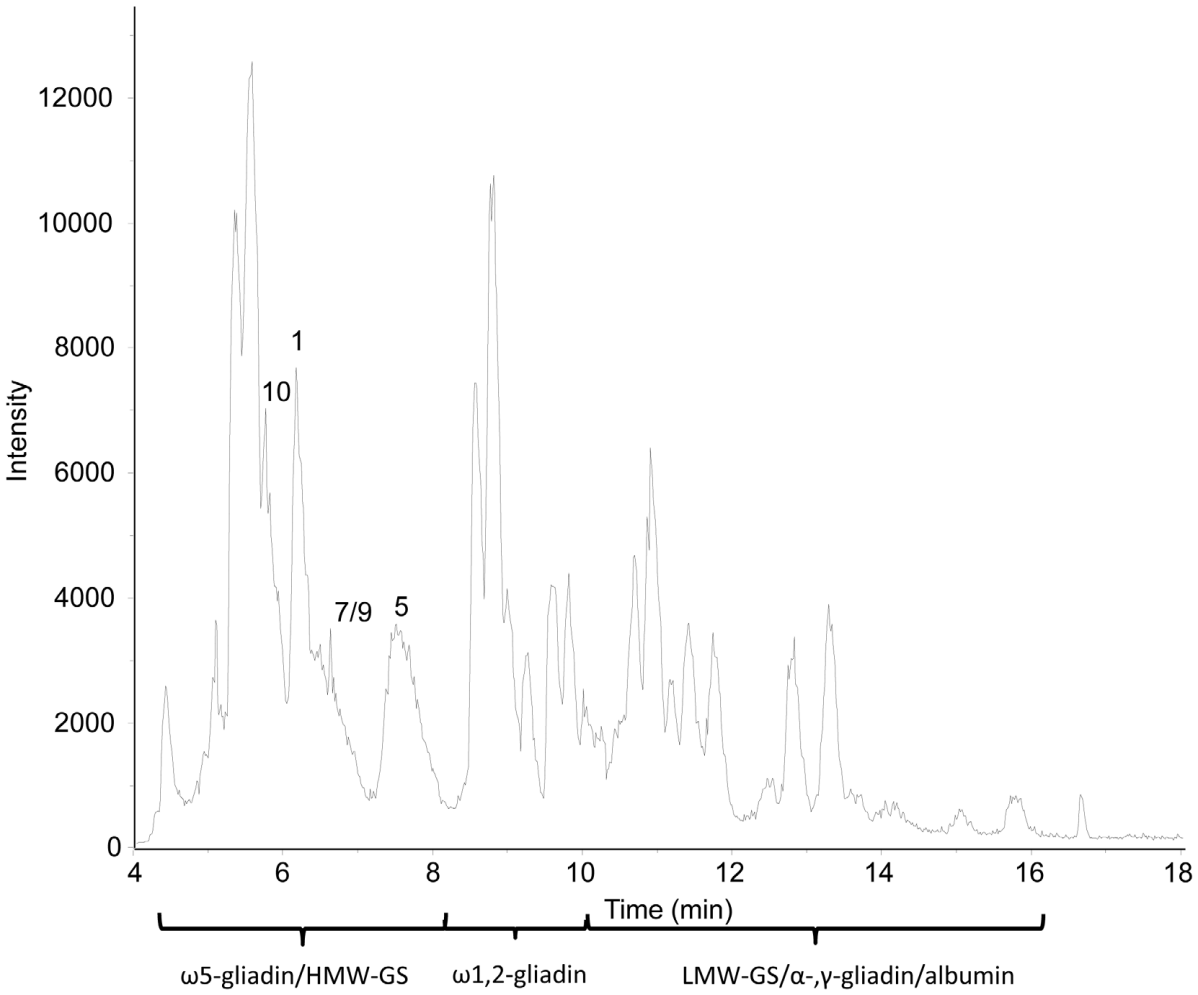
Base peak chromatogram from RP-HPLC-ESI-MS of the isolated HMW-GS fraction of wheat cv. Akteur. The mobile phase contains 0.1% (v/v) formic acid. HMW-GS are labeled with numbers according to the current nomenclature system [4].

Figure 5 shows the BPC of the dissolved HMW-GS fraction of cv. Akteur after LC-MS using the C_4_ column under the same conditions as in the previous measurements, except that the mobile phase contained 0.1% (v/v) formic acid instead of 0.1% (v/v) TFA. Both the overall peak intensities as well as the number of peaks were increased (Figure 5). To determine the exact retention order of gluten proteins when separated in the presence of formic acid, mass spectra of every peak in the BPC were deconvoluted into protein M_r_s. The detected M_r_s in the isolated HMW-GS fraction of cv. Akteur are given in Table 4. When comparing Table 2 to 4, it is clear that more and other types of proteins were distinguished with formic acid instead of TFA in the mobile phase. Additional ω5- and ω1,2-gliadins were identified based on their M_r_ and RT. Other proteins were detected in the time interval associated with the RT of LMW-GS, α-, and γ-gliadins [22], and proteins were identified accordingly (Table 4). Also non-gluten proteins, such as α-amylase-inhibiting proteins, were recognized (Table 4). All these proteins formed the trace fraction (<4%) of other wheat proteins that co-precipitated during the isolation of HMW-GS (Table 1) and could not be detected with SDS-PAGE (Figure 1). With formic acid in the eluent, the sensitivity of the MS detection clearly increased. This was also underlined by improved mass intensities of the detected proteins in the BPC. For example a twofold increase of intensity for the ω5-type gliadins and even a 3-fold increase of intensity for HMW-GS Dx5 were observed (Figures 3 and 5).

**Table 4:** Relative average molecular masses (M_r_) and corresponding proteins present in the isolated HMW-GS fractions of the cv. Akteur as determined by RP-HPLC-ESI-MS with the mobile phase containing 0.1% (v/v) formic acid.

RT (min)	M_r_ 1	Tentative identification^2^
5.4	48,871 (0.4)	ω5-gliadin
5.6	54,835 (0.6)	ω5-gliadin
5.8	54,950 (0.6)	ω5-gliadin
	67,476 (0.6)	HMW-GS Dy10
6.2	51,527 (0.4)	ω5-gliadin
	87,697 (0.7)	HMW-GS Ax1
6.6 (6.5–7.2)	82,529 (0.7)	HMW-GS Bx7
	73,519 (0.5)	HMW-GS By9
7.5 (7.3–8.1)	88,114 (0.8)	HMW-GS Dx5
8.6	42,744 (0.2)	ω1,2-gliadin
8.8	41,862 (0.4)	ω1,2-gliadin
9.0	13,275 (0.2)	α-amylase inhibitor
9.3	40,891 (0.4)	ω1,2-gliadin
9.6	41,834 (0.6)	ω1,2-gliadin
9.8	40,953 (0.6)	ω1,2-gliadin
10.0	30,175 (0.6)	LMW-GS
10.7	39,336 (0.4)	LMW-GS
10.9	13,335 (0.2)	α-amylase inhibitor
11.2	34,004 (0.6)	α-gliadin
11.3–12	13,190 (0.3)	α-amylase inhibitor
12.8	35,196 (0.5)	γ-gliadin
13.3	38,645 (0.5)	γ-gliadin
15.8	31,051 (0.6)	γ-gliadin

1 Standard deviations are given between brackets.

2 Based on M_r_ agreement with proteins from the wheat storage proteome [5], [23], [24], [36].

**Figure 6:**
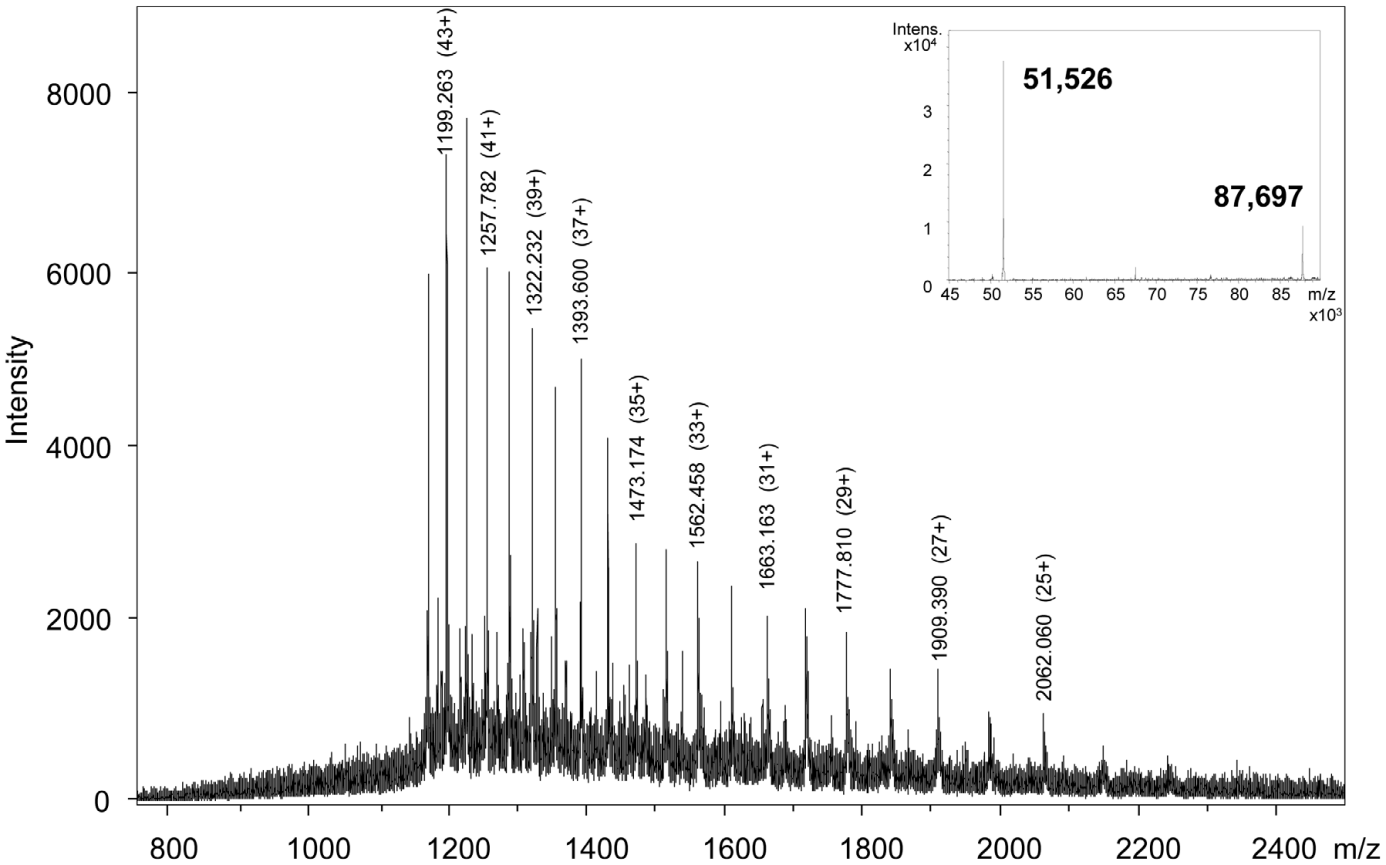
Mass spectra of wheat HMW-GS Ax1 and ω5-gliadin. The spectra are taken from the average of scans under the peak with retention time 6.2 min from the base peak mass chromatogram after RP-HPLC-ESI-MS with 0.1% (v/v) formic acid in the mobile phase. The simulated mass spectra obtained by maximum entropy deconvolution are shown as insets.

Nevertheless, the chromatographic separation of HMW-GS was worse when replacing TFA by formic acid in the mobile phase. Only subunit 5 appeared as a (broad) separate peak in the BPC (RT 7.3–8.1 in Figure 5). Subunits 7 and 9 co-eluted without forming clearly separated peaks in the BPC (RT 6.5–7.2 in Figure 5), whereas the elution range of subunits 1 and 10 overlapped with that of ω5-gliadins (Table 4). Due to this overlap, the identification of subunits 1 and 10 from the BPC with related-ion deconvolution was difficult, because their MS signals were suppressed by those of the ω5-type gliadins which had higher MS intensities. Entropic deconvolution of the mass spectra taken from the average of scans under the peaks of the ω5-gliadins revealed the location of the missing subunits, as illustrated in Figure 6. Similar observations were made for cvs. Contra and Apache, for which only subunits 2 and 3, respectively, could be identified in a separate peak. The other subunits also overlapped with the ω5- gliadins (results not shown). Despite the higher signal intensities, the chromatographic separation without the ion-pairing agent TFA was not sufficient to distinguish all HMW-GS separate from the other gluten protein types. Although the HMW-GS purity after isolation was high, the high signal intensities of other proteins present in low amounts interfered with the spectra of HMW-GS when no TFA was present in the mobile phase. Apparently, the excellent ion-pairing characteristics of TFA in the mobile phase were necessary, not only to separate the HMW-GS from the other wheat proteins, but also to separate the individual subunits from each other.

### Conclusions

Intact HMW-GS can be identified from the wheat proteome by ESI-MS after isolation from flour, dissolution in water/ACN, and separation by RP-HPLC. A high resolution MS is required to obtain reliable values for the Mr of the proteins after deconvolution. TFA rather than formic acid should be used in the mobile phase, because good chromatographic separation is a prerequisite to detect HMW-GS without interference of other protein types. Although they form only a minor fraction in HMW-GS isolates, but also in the wheat storage proteome, ω-gliadins show a high response in LC-MS and can be detected with high sensitivity. Altogether, to detect HMW-GS from the wheat proteome, it is necessary to isolate or enrich HMW-GS from wheat flour beforehand to reach sufficient signal intensity in MS and to avoid overlap with other wheat proteins during RP-HPLC. The present MS-compatible separation of intact HMW-GS allows MS fragmentation and fragment separation of each protein individually and, hence, offers a basis for further top-down proteomics of the wheat storage proteome.
